# Supplementary material for: Acceptability and perceived barriers to adoption of the core outcome set for maternal and neonatal health research and surveillance during emerging and ongoing epidemic threats (MNH-EPI-COS): An online survey
Source: PLOS Glob Public Health. 2025 Nov 25;5(11):e0005474. doi: 10.1371/journal.pgph.0005474 (PMC12646480; doi:10.1371/journal.pgph.0005474)
Supplement: S2 File — (DOCX) [file pgph.0005474.s002.docx]

# S2 File

Summary of the methods of previous phases

**Final consensus on MNH-EPI-COS**

N

**MNH-EPI-COS DEVELOPMENT PROCESS**

**Systematic review**

N

**Consensus meetings** (online, in-person)

n = 24

N

**Delphi round 2** (online)

n = 141 / 150 (91 %)

N

**ACCEPTABILITY SURVEY**

**Online consultation**

n = 100/118 (85%)

- Acceptability of the final MNH-EPI-COS
- Acceptability of the definitions
- Anticipated barriers to COS adoption
- Perceived feasibility of data collection

**Delphi round 1** (online)

n = 150 / 197 invited (76%)

N
