# Supplementary material for: Acceptability and perceived barriers to adoption of the core outcome set for maternal and neonatal health research and surveillance during emerging and ongoing epidemic threats (MNH-EPI-COS): An online survey
Source: PLOS Glob Public Health. 2025 Nov 25;5(11):e0005474. doi: 10.1371/journal.pgph.0005474 (PMC12646480; doi:10.1371/journal.pgph.0005474)
Supplement: Attachment — Submitted filename: 20250715_Point-by-point response.docx [file pgph.0005474.s007.docx]

**POINT-BY-POINT RESPONSE TO REVIEWERS' COMMENTS**

**Manuscript reference: PGPH-D-25-00952**

**Title: Acceptability and perceived barriers to adoption of the core outcome set for maternal and neonatal health research and surveillance during emerging and ongoing epidemic threats (MNH-EPI-COS). An online survey**

**PLOS Global Public Health**

| **Reviewer** | **Reviewer Comment** | **Response** |
| --- | --- | --- |
| **Journal editor** | 1. We have amended your Competing Interest statement to comply with journal style. We kindly ask that you double check the statement and let us know if anything is incorrect. | Yes, thank you for the amendment. We have reviewed the revised Competing Interest statement and confirm that it is correct. |
| **Journal editor** | 2. In the online submission form, you indicated that Data are available upon reasonable request.  All PLOS journals now require all data underlying the findings described in their manuscript to be freely available to other researchers, either  1. In a public repository,  2. Within the manuscript itself, or  3. Uploaded as supplementary information.  This policy applies to all data except where public deposition would breach compliance with the protocol approved by your research ethics board. If your data cannot be made publicly available for ethical or legal reasons (e.g., public availability would compromise patient privacy), please explain your reasons by return email and your exemption request will be escalated to the editor for approval. Your exemption request will be handled independently and will not hold up the peer review process, but will need to be resolved should your manuscript be accepted for publication. One of the Editorial team will then be in touch if there are any issues. | Thank you for this clarification. We now make the underlying dataset publicly available as an **Excel file in the Supplementary Material** to comply with PLOS data policy.  . |
| **Journal editor** | 3. We do not publish any copyright or trademark symbols that usually accompany proprietary names, eg (R), (C), or TM  (e.g. next to drug or reagent names). Please remove all instances of trademark/copyright symbols throughout the text, including ™ on page 8. | Thank you for your note. We have removed all trademark and copyright symbols throughout the manuscript, including the ™ on page 8. |
| **Reviewer #1** | Dear authors, The study provides valuable insights into COS acceptability but requires stronger methodological rigor, real-world validation, and actionable implementation strategies. Addressing these critiques would enhance its impact and applicability across diverse settings. Accordingly, please respond to the following: 1.The study participants were limited to stakeholders involved in the earlier phases of the MNH-EPI-COS development, potentially introducing selection bias. The absence of new or independent stakeholders may skew the results toward pre-existing biases. Including a broader range of stakeholders, such as frontline healthcare workers and policymakers not previously involved in the COS development, would eventually validate the findings more robustly. | We sincerely appreciate this insightful comment, which aligns closely with the next phase of our work. In this study, we focused our assessment on stakeholders who participated directly in the first round of the MNH-EPI-COS development process and who were not part of the small group that reached the final consensus on the COS. There is limited literature on the acceptability of COS among broader stakeholder groups, as finalization typically occurs within these smaller groups. While this approach allowed us to capture informed perspectives on the finalized COS, we recognize that it does not allow for an evaluation of its acceptability among new or independent stakeholders. We were aware of this limitation and have revised the abstract and introduction to clarify the scope of the acceptability assessment, and we have reinforced this point explicitly in the limitations section of the manuscript.  The results included in this manuscript were part of a step within the MNH-EPI-COS development initiative that did not include validation beyond the expert panel. As additional context, we would like to share that we are currently working on follow-up studies aimed at broadening stakeholder engagement and strengthening the real-world applicability of the outcomes.  We are currently implementing a follow-up study funded by the Gates Foundation, which includes:   - Development and evaluation of a data collection toolkit: We are creating practical resources to support the integration of the MNH-EPI-COS into maternal and neonatal health research and surveillance. This toolkit will standardize data harmonization and analysis across diverse settings, with a focus on health emergency contexts. - End-user validation: Through pilot-testing with research and epidemiological teams, we will: - Retrospectively extract data from existing databases on infectious diseases and maternal/perinatal health during outbreaks; - Conduct semi-structured questionnaires and virtual consultations to gather feedback from stakeholders not involved in the initial COS development. |
| **Reviewer #1** | 2.The survey items were not based on a systematic literature review, which could lead to gaps in addressing all relevant barriers and acceptability factors. Please conduct a systematic review to identify all potential barriers and facilitators of COS adoption, ensuring the survey comprehensively covers these aspects. | We sincerely appreciate this thoughtful point regarding methodological rigor. While a full systematic review would indeed be ideal, resources and timeline constraints precluded this step during survey development. To ensure comprehensive coverage despite this limitation, explicitly acknowledged in our *Limitations section,* we conducted an exhaustive literature review, detailed on page 6, that included:   1. Peer-reviewed publications on COS uptake barriers and facilitators (referenced in our paper as citations 9,11–16,20), 2. Validated frameworks for measuring acceptability (citations 17–19).   This synthesis allowed us to identify critical domains while working within project constraints. We fully agree that a systematic review remains the gold standard and will prioritize this approach in future work to strengthen generalizability. |
| **Reviewer #1** | 3.The feasibility of data collection was assessed hypothetically, as the COS had not yet been implemented. This may not accurately reflect real-world challenges. It was better to pilot the COS in a few diverse settings to gather empirical data on feasibility and barriers before finalizing the survey results. I suggest marking this note as a limitation of your work. | Thank you for your suggestion. We have reinforced this point in the discussion and the limitations section, emphasizing the need to validate anticipated barriers through piloting the COS in real-world settings across diverse contexts.  As mentioned in a previous response, pilot testing of the COS with research and epidemiological teams is currently underway and is expected to provide insights into potential implementation barriers in real-world scenarios. |
| **Reviewer #1** | 4.The qualitative analysis of open-ended responses lacks detail on methodology (e.g., coding framework, inter-rater reliability), raising concerns about the rigor and reproducibility of the findings. Please provide a detailed description of the qualitative analysis process, including how themes were identified and validated, to enhance transparency. | Thank you for your valuable comment. We have revised the manuscript to provide a clearer and more detailed description of the qualitative analysis process in Material and methods section. Specifically, we now clarify that open-ended responses were coded and analyzed separately for each survey question using inductive thematic analysis. Two independent coders developed question-specific coding frameworks, and any discrepancies were resolved through discussion and consensus. Emerging themes were then reviewed with the broader research team to enhance interpretive rigor, address potential bias, and strengthen the credibility of the findings. |
| **Reviewer #1** | 5.Many outcomes (e.g., "maternal confirmed infection," "congenital anomalies") were deemed less feasible in low-resource settings due to limited infrastructure and training. This is another limitation to be documented at the end of your work. | We sincerely thank you for this important observation, which aligns with one of our most significant findings. The feasibility concerns for certain outcomes in low-resource settings were indeed a central focus of our analysis. Rather than a limitation, we view this as a critical strength of our study: by systematically identifying these implementation barriers early, we can directly address them through our next steps in this project, as previously detailed and now further elaborated in the updated Discussion section. |
| **Reviewer #1** | 6.The paper acknowledges civil society voices were marginalized in consensus meetings but does not explore how this might affect COS adoption or equity. I encourage you to include a deeper analysis of power dynamics and strategies to amplify underrepresented voices (e.g., community engagement frameworks). | Thank you for this important comment. Throughout the MNH-EPI-COS process—including both the initial development phase and the current acceptability study—we actively invited representatives from civil society organizations. However, including civil society voices in consensus processes presents well-documented challenges, particularly the risk that their perspectives may be marginalized or diluted.  Aware that discussions dominated by professionals could undermine patient and community perspectives, we took specific actions during the development phase to address this risk. Notably, we organized an independent virtual consultation with civil society representatives to ensure their views could be shared freely, without professional influence. This approach aligns with recommendations from the RAND Corporation and COS developers, who propose holding separate deliberations with civil society stakeholders before integrating their input into the broader consensus process. These steps are described in detail in the main paper outlining the MNH-EPI-COS development phase (1).  In the current acceptability study, we observed that some of the same patient-oriented outcomes that had been highly valued by civil society participants during the development phase continued to receive lower levels of endorsement within the general panel. We have revised the discussion to clarify that the power dynamics identified during development may persist into later stages and influence how certain outcomes are perceived and prioritized. We also reflect on the broader equity implications and emphasize the importance of structured strategies—such as independent consultations and community engagement frameworks—to ensure the meaningful and sustained inclusion of underrepresented voices throughout the entire COS process. |
| **Reviewer #1** | 7.The study does not address how the COS will be updated or maintained over time to remain relevant. Based on that, recommend a governance structure (e.g., steering committee) to review and update the COS periodically based on new evidence and stakeholder feedback. | We sincerely thank the reviewer for this valuable comment and fully agree that a governance structure is essential for maintaining the COS’s relevance. We have addressed this point in the Discussion section and would like to amplify it here by highlighting two key ongoing actions:   1. Additional COS evaluations are currently underway, including:    - Pilot testing using semi-structured questionnaires and virtual consultations to gather critical feedback from stakeholders not involved in the initial COS development;    - Evaluation of implementation outcomes (acceptability, feasibility, and perceived usefulness of the MNH-EPI-COS) among maternal/newborn health stakeholders in outbreak contexts;    - Synthesis of barriers, facilitators, and contextual factors affecting MNH-EPI-COS implementation in recent/ongoing studies. 2. Active steering committee: Our pre-existing committee (which oversaw the study conduct and remains engaged) will be formally tasked with periodically evaluating the need for COS updates. |
| **Reviewer #1** | 8.Ethical implications of excluding certain populations (e.g., non-English speakers, rural communities) from the survey are not discussed. I invite you to address ethical gaps by ensuring future surveys are multilingual and inclusive of marginalized groups to enhance equity. | We sincerely appreciate this crucial ethical observation. While our initial COS development stakeholders included Spanish- and English-speaking participants with diverse professional backgrounds, geographic regions, and gender representation, we fully acknowledge the insufficient inclusion of marginalized populations and non-English speakers. As explicitly noted in our Limitations section, we are now addressing this gap through dedicated initiatives in our ongoing project, including deliberate engagement strategies for multilingual and underserved communities. |
| **Reviewer #2** | The study titled Acceptability and perceived barriers to adoption of the core outcome set for maternal and neonatal health research and surveillance during emerging and ongoing epidemic threats (MNH-EPI-COS). An online survey Understanding the acceptability and barriers to adopting the core outcome set for maternal and neonatal health during epidemics (MNH-EPI-COS) is critical for effective global health responses. This research identifies perceived challenges that could prevent adoption of core outcome set of standardized data collection (for example during crises like COVID-19), potentially hampering evidence-based decision-making and cross-study comparison. This paper is connected to the published article of the same research team: “A core outcome set for maternal and neonatal health research and surveillance of emerging and ongoing epidemic threats (MNH-EPI-COS): a modified Delphi-based international consensus; Pingray, Verónica et al.; eClinicalMedicine, Volume 80, 103025” I am recommending publishing this manuscript with minor changes. | We sincerely appreciate your thorough review and valuable feedback. We have implemented all suggested revisions as detailed below and believe they significantly strengthen the manuscript. Thank you for your time and expertise. |
| **Reviewer #2** | Abstract: It is understandable that MNH-EPI-COS is prominent acronym, however, author can elaborate it first time in the abstract. | We agree that clarity on the COS name is essential. MNH-EPI-COS denotes a Core Outcome Set designed specifically for Maternal and Neonatal Health research during EPIdemics. We clarified this in the abstract. |
| **Reviewer #2** | Analysis: Line 159: Authors can more elaborate on the process of qualitative analysis | Thank you for your comment. We have expanded the description of the qualitative analysis process in the revised manuscript, providing more detail on how the data were coded, how themes were developed, and the steps taken to ensure rigor and reliability in the analysis. |
| **Reviewer #2** | References: Line 354: Authors could provide DOI or PMID number of all references. | Thank you for your suggestion. We have now added the DOI or PMID numbers to all references in the revised manuscript. |
| **Reviewer #2** | Conclusion Line 339: Authors can re-suggest in the conclusion as “Recommendation for development of toolkit is important for adoption and implementation”. | Thank you for your suggestion. We have revised the conclusion to emphasize that the development of a practical implementation toolkit is important to support the adoption and implementation of the MNH-EPI-COS. |
| **Reviewer #2** | -No information on the data availability and not as per the requirement of PLOS data availability policy. | Thanks for this comment. We now make the underlying dataset publicly available as a Excel File in the **Supplementary Material** (**Supplementary File SF5**) to comply with PLOS data policy. |

1.	Pingray V, Klein K, Alonso JP, Belizan M, Babinska M, Alger J, et al. A core outcome set for maternal and neonatal health research and surveillance of emerging and ongoing epidemic threats (MNH-EPI-COS): a modified Delphi-based international consensus. EClinicalMedicine. 2025 Feb;80(103025):103025.
